# Thermally Induced Osteocyte Damage Initiates a Remodelling Signaling Cascade

**DOI:** 10.1371/journal.pone.0119652

**Published:** 2015-03-18

**Authors:** Eimear B. Dolan, Matthew G. Haugh, Muriel C. Voisin, David Tallon, Laoise M. McNamara

**Affiliations:** 1 Biomechanics Research Centre (BMEC), Biomedical Engineering, College of Engineering and Informatics, National University of Ireland, Galway, Ireland; 2 National Centre for Biomedical Engineering Science (NCBES), National University of Ireland, Galway, Ireland; 3 Stryker Ireland, Carrigtwohill, Cork, Ireland; Universidade Federal do Rio de Janeiro, BRAZIL

## Abstract

Thermal elevations experienced by bone during orthopaedic procedures, such as cutting and drilling, exothermal reactions from bone cement, and thermal therapies such as tumor ablation, can result in thermal damage leading to death of native bone cells (osteocytes, osteoblasts, osteoclasts and mesenchymal stem cells). Osteocytes are believed to be the orchestrators of bone remodeling, which recruit nearby osteoclast and osteoblasts to control resorption and bone growth in response to mechanical stimuli and physical damage. However, whether heat-induced osteocyte damage can directly elicit bone remodelling has yet to be determined. This study establishes the link between osteocyte thermal damage and the remodeling cascade. We show that osteocytes directly exposed to thermal elevations (47°C for 1 minute) become significantly apoptotic and alter the expression of osteogenic genes (*Opg* and *Cox2*). The *Rankl/Opg* ratio is consistently down-regulated, at days 1, 3 and 7 in MLO-Y4s heat-treated to 47°C for 1 minute. Additionally, the pro-osteoblastogenic signaling marker *Cox2* is significantly up-regulated in heat-treated MLO-Y4s by day 7. Furthermore, secreted factors from heat-treated MLO-Y4s administered to MSCs using a novel co-culture system are shown to activate pre-osteoblastic MSCs to increase production of the pro-osteoblastic differentiation marker, alkaline phosphatase (day 7, 14), and calcium deposition (day 21). Most interestingly, an initial pro-osteoclastogenic signaling response (increase *Rankl* and *Rankl/Opg* ratio at day 1) followed by later stage pro-osteoblastogenic signaling (down-regulation in *Rankl* and the *Rankl/Opg* ratio and an up-regulation in *Opg* and *Cox2* by day 7) was observed in non-heat-treated MLO-Y4s in co-culture when these were exposed to the biochemicals produced by heat-treated MLO-Y4s. Taken together, these results elucidate the vital role of osteocytes in detecting and responding to thermal damage by means of thermally induced apoptosis followed by a cascade of remodelling responses.

## Introduction

During orthopedic intervention, bone and the surrounding soft tissue can be exposed to elevated temperatures arising from surgical cutting and drilling, exothermal reactions from bone cement during curing, and thermal therapies such as tumor ablation. Temperatures up to 100°C have been reported to arise in surrounding bone tissue as a result of the cutting and drilling processes during orthopaedic surgeries [1,2,3]. Polymethylmethacrylate polymerisation can result in temperatures ranging from 40 to 100°C [4,5]. Thermal ablation has been widely used treat a number of bone diseases including malignant bone tumors [6] and bone metastasis [7], whereby temperatures >48°C are utilised to kill the cancer cells. Additionally, using experimentally informed computational methods, we have predicted in a previous study that embedded osteocytes immediately experience the thermal elevations of the surrounding matrix in which they are entombed [8].

Temperature elevations trigger responses at the cellular level, such as cellular apoptosis and necrosis, which in turn lead to organ level reactions, the extent of which is dependent on the temperature itself and the duration of exposure [9,10,11]. Our recent study has shown that in vitro exposure of bone cells to temperatures exceeding 45°C can trigger cellular responses, such as necrosis and apoptosis, depending on the extent and duration of thermal exposure and the phenotype of the cell [11]. Specifically, we showed that osteocyte-like cells are more resilient to heat-induced cellular death than osteoblast-like cells, whereby a large apoptotic response was observed at 12, 24 hours and 4 days after osteocyte-like MLO-Y4 cells were exposed to elevated temperatures (≥45°C), and this apoptotic response occurred to a lesser extent in osteoblast-like MC3T3-E1 cells. When thermal elevation was minimised to 45°C for 1 minute MLO-Y4 cells completely recovered by 4 days (as indicated by percentage necrosis, viability and population size), whereas osteoblast-like MC3T3-E1 cells could only withstand the same temperature for 30 seconds.

Additionally, mild thermal elevations (42.5–44°C) have been shown to stimulate bone remodelling, trabecular bone formation and increase cortical bone density of the rabbit femur after undergoing surgical trauma [12]. Direct exposure of osteoprogenitor cells to mild thermal elevations (39–42.5°C) for extended durations (≥1 hour) induced differentiation along the osteoblastic lineage and enhanced mineralised nodule formation in vitro [13]. Interestingly, we have recently shown that markers related to osteogenesis (alkaline phosphatase activity and calcium deposition) were up-regulated in Mesenchymal Stem Cells (MSCs) when directly exposed to clinically relevant elevated temperatures ≥47°C for much shorter durations (30 seconds) in vitro [8,11]. It is known that soluble factors secreted by mechanically stimulated osteocytes (conditioned media) significantly up-regulated the expression of osteogenic genes osteopontin and *Cox2* by MSCs in vitro [14]. Moreover, the percentage of alkaline phosphatase labelled surfaces and bone formation rate were significantly increased in human trabecular bone ex vivo in response to mechanical stimulation [15].

Surgically induced matrix damage and fatigue induced micro-damage to bone are well understood initiators of osteocyte apoptosis in vivo [16,17,18,19,20]. Most recently, it has been shown that osteocyte apoptosis directly results in an up-regulation of pro-osteoclastic signaling markers by nearby healthy cells, and thereby initiates the repair response [17,21,22]. Such studies provide strong evidence that site-specific osteocyte apoptosis is the underlying mechanism by which targeted removal of damaged bone tissue by osteoclastic bone resorption is initiated. Osteocytes have been identified as the major orchestrators of skeletal activity, sensing mechanical and chemical stimuli to regulate bone remodeling by directly influencing nearby cells [23,24]. However, whether thermally induced osteocyte apoptosis in vitro can initiate a similar signalling response as micro-damage induced osteocyte apoptosis remains to be determined.

There is a distinct lack of knowledge regarding the mechanisms by which bone cells respond to thermal elevations associated with orthopaedic procedures and as such, initiate the remodelling process. The aim of this study is to establish the link between osteocyte thermal damage and the signalling mechanisms leading to bone remodelling in vitro. Osteocyte-like MLO-Y4s were exposed to thermal elevations of 47°C for 1 minute, which we have shown to be likely to occur during orthopaedic procedures [8]. Next, secreted factors from these heat-treated MLO-Y4 cells were administered to non-heat-treated MLO-Y4 cells using a novel co-culture system. This co-culture system allowed non-heat-treated MLO-Y4s to be exposed to biochemical molecules produced by the heat-treated MLO-Y4’s, without directly undergoing heat-treatment themselves. mRNA from the heat-treated and the co-cultured osteocyte-like MLO-Y4 cells was analysed by real time polymerase chain reaction (RT-PCR) for the expression of key signalling factors related to the activation of osteoclastic differentiation (*Rankl* and *Opg*) and osteoblastic differentiation (*Cox2*). Furthermore, using the same co-culture technique secreted factors from the heat-treated MLO-Y4 cells were administered to Balb/c MSCs. ALP expression and mineralisation were analysed in the Balb/c MSCs co-cultured with heat-treated MLO-Y4s to confirm osteoblastic differentiation.

## Materials and Methods

### Cell Culture

Osteocyte-like MLO-Y4 cell line and Balb/c mouse MSCs were used in this study. Murine-derived MLO-Y4 cells (a gift from Prof. Lynda Bonewald, San Antonio [25]) have many similar characteristics with primary osteocytes such as low alkaline phosphatase (ALP) expression, high osteocalcin production and numerous dendritic processes [25] and have been widely used in the study of osteocyte biology [11,21,26,27,28]. MLO-Y4 cells were cultured on collagen-coated flasks in α-modified minimal essential medium (α-MEM) supplemented with 2.5% foetal bovine serum (FBS, HyClone Laboratories Inc.), 2.5% iron-supplemented foetal calf serum (FCS, HyClone Laboratories Inc.), 2 mM L-glutamine, 100 U/mL penicillin and 100mg/mL streptomycin.

Balb/c primary MSC cultures characterised according to the protocols of Peister et al. [29] were obtained in our laboratory as previously described [11,30,31], under ethical approval from the Animal Care Research Ethics (ACREC) committee at the National University of Ireland, Galway, and under licence from the Irish Department of Health and Children and in compliance with the Council of European Union directive 86/609. Briefly, tibiae and femora were removed from 8–10 week old mice (female and male) and cultured in RPMI-1640 medium supplemented with 9% FBS, 9% horse serum (HS), 100 U/mL penicillin, 100 g/mL streptomycin and 2 M L-glutamine. Bones were clipped at the ends, and centrifuged at 400g for 2 min. The cell pellets were plated onto T175 flasks for 24 hours, the flasks were then washed with sterile phosphate-buffered solution (PBS). Media was replenished and MSC cells were cultured until large colonies were observed (approximately 4 days). The cells were then re-plated and cultured for an additional 10 days. Balb/c MSCs were maintained in Iscoves Modified Eagles Medium (MEM) supplemented with 10% FBS, 10% HS, 2 mM L-glutamine, 100 U/mL penicillin and 100 mg/mL streptomycin until confluent. The osteogenic, chondrogenic and adipogenic potential of these cells was confirmed as previously described [30]. All cells were maintained in 75 cm^2^ tissue culture flasks at 37°C in a 5% CO_2_ humidified atmosphere. Once confluent, cells were detached using trypsin–EDTA, seeded at a density of 10^4^ cells/cm^2^ and pre-incubated for 24 hours prior to heat exposure experiments. All chemicals and reagents were purchased from Sigma-Aldrich, unless otherwise stated.

### Heat exposure experiments

MLO-Y4 cells were exposed to preheated media at 47°C or 37°C (control), and maintained on a hot plate at these temperatures for 1 minute as previously described [11]. Briefly, the temperature accuracy of the hot plate is ±0.3°C, and the temperature distribution uniformity on the surface of the plate is ±0.2°C. A thermocouple was inserted into an unused well to monitor the temperature of the preheated media, and this temperature measurement was accurate to ±1°C for the duration of heat exposure. After exposure to the elevated temperatures MLO-Y4’s were returned to a CO_2_ incubator for 30 minutes to allow the cells to reach to the normal cell culture temperature of 37°C. Heat-treated MLO-Y4’s were then either cultured for recovery periods of 1 day, for fluorescent microscopy and flow cytometry analysis, up to 7 days, for real time polymerase chain reaction (RT-PCR) analysis, or used for further co-culture experiments described below.

### Fluorescent Microscopy

MLO-Y4s were fixed 24 hours after heat-treatment in 4% paraformaldehyde for 15 minutes and permeabilised in 0.1% Triton X-100 for 5 minutes. The actin cytoskeleton was stained by incubating the cell in PBS containing 1% fluorescein isothiocyanate-conjugated phalloidin for 45 minutes at room temperature. The cover-slips were mounted with Vectashield mounting media with 4,6-Diamidino-2-phenylindole (DAPI) nuclear counter stain. Fluorescence was visualised with an Olympus BX51 Upright Fluorescent Microscope at 20X magnification.

### Flow Cytometry

Percentage apoptosis, necrosis and viability in the heat-treated MLO-Y4 cell population was determined using a flow cytometry technique (BD FACS CANTO) by staining with propidium iodide (PI) and Annexin V-fluorescein isothiocyanate (FITC) (ImmunoTools GmbH, Germany) as described in a previous study [11]. Briefly, PI stains cells that have lost membrane integrity, a feature of necrosis, whereas Annexin V-FITC stains phosphatidylserine (PS), a phospholipid that translocates from inside to outside the lipid membrane during apoptosis. Live cells are double negative for Annexin V-FITC and PI, apoptotic cells are Annexin V-FITC positive and PI negative, and advanced apoptotic and necrotic cells are positive for both Annexin V-FITC and PI. Floating cells were collected and adherent cells were detached by incubation with Trypsin-EDTA for 2 minutes. Cells were washed and re-suspended in 500 μL of Annexin V Buffer (10 mM HEPES, 150 mM NaCl, 5 mM KCL, 1 mM MgCl2, 1.8 mM CaCl2) [32]. Annexin V-FITC (7 μg/mL) was added and samples were incubated for 15 minutes on ice and in the dark. Subsequently, 4 μL of 20 μg/mL PI was added to each sample, vortexed briefly. Stained cells were subjected to flow cytometric analysis. Annexin V staining was detected in the FL1 channel and propidium iodide was detected in the FL2 channel. A minimum of 10,000 events were acquired and cell debris was excluded from analysis by appropriate forward and right angle scatter threshold settings.

### MLO-Y4 Co-culture experiments

Non-heat-treated MLO-Y4 cells were cultured on permeable inserts (PET 1 μm pores; Millipore, Cork, Ireland) at a density of 10^4^ cells/cm^2^ for 24 hours prior to addition to the wells of heat-treated MLO-Y4s and co-cultured for up to 7 days. The permeable inserts ensured that ccMLO-Y4s were physically separated from the heat-treated MLO-Y4 cells, thereby preventing direct cell-to-cell contact but allowing ccMLO-Y4s to be exposed to soluble factors produced by the heat-treated MLO-Y4 cells, without undergoing heat-treatment themselves. At each media change, half of the spent media was carefully replenished in each well so as not to remove all of the secreted biochemicals and cellular debris produced by the heat-treated cells. As such, the cells were continually exposed to the biochemical factors induced by heat-treatment. After 7 days ccMLO-Y4s were analysed by RT-PCR for the expression of key signalling factors related to osteoclastic and osteoblastic differentiation.

### Gene Expression

mRNA from the heat-treated and the co-cultured MLO-Y4 cells was analysed by real time polymerase chain reaction (RT-PCR) for the expression of key signalling factors related to the activation of osteoclast differentiation (Receptor activator of nuclear factor kappa-B ligand (*Rankl*) and osteoprotegerin (*Opg*), and pro-osteoblastic differentiation (Cyclooxygenase 2 (*Cox2*)). Glyceraldehyde 3-phosphate dehydrogenase (*Gapdh*) was used as a housekeeping gene. *Rankl* has been widely shown to be a necessary factor for osteoclastogenesis [33,34]. Osteoclast differentiation is inhibited by the *Rankl* inhibitor *Opg*, a soluble decoy receptor for *Rankl* [35,36]. The *Rankl* /*Opg* ratio is an important ratio governing osteoclast activity, an increase promotes osteoclast differentiation while a decrease reduces mature osteoclast concentration [36,37,38,39]. *Cox2* has also been shown to be vital signalling factor for osteogenic differentiation [40,41].

Cells were lysed using 700 μL lysis buffer and a cell scraper was used to ensure cells were detached. The solution was homogenised and the RNA was precipitated using 70% ethanol. The RNA was then washed using an ENZA RNA isolation kit according to the manufacturer's protocol (Omega Bio-tek) and dissolved in 40 μL of RNAse free water (Qiagen). The quality of the RNA was measured using a Nanodrop spectrometer (Thermo-scientific) before being converted to cDNA using a cDNA synthesis kit (Omega Biosciences) and Gene Amp 9700 A Thermal cycler (Applied Biosystems). RT-PCR was performed on a Step-One plus analyser (Applied Biosystems) using Taqman probes (Applied Biosystems) for *Rankl* (Mm00441906_m1), *Opg* (Mm00435452_m1) and *Cox2* (Mm00478374_m1). RT-PCR data was analysed using the 2^−ΔΔCt^ method [42], with *Gapdh* (Mm99999915_g1) as a housekeeping gene. All gene expression experiments were conducted in triplicate.

### Balb/c MSC Co-culture experiments

For Balb/c MSC co-culture experiments, non-heat-treated Balb/c MSCs on inserts were added to the wells of the heat-treated MLO-Y4s and co-cultured for up to 21 days. Balb/c MSCs (ccMSCs) were cultured on permeable inserts (PET 1 μm pores; Millipore, Cork, Ireland) at a density of 10^4^ cells/cm^2^ and cultured for 24 hours prior to addition to the heat-treated MLO-Y4 cells. After 21 days in co-culture, the pro-osteoblastic differentiation marker, alkaline phosphatase, and calcium deposition were analysed to investigate activation of the ccMSCs.

Intracellular alkaline phosphatase (ALP) activity of ccMSCs was measured after 21 days using cell lysate measured colorimetrically with p-nitrophenyl phosphate as a substrate (Sigma). ALP expression indicates differentiation along the osteoblastic lineage and is an early marker of mineralisation. To quantify membrane-bound ALP, cell lysate was prepared from ccMSCs using three cycles of freeze- thaw in deionized distilled water. 40 μL of cell lysate was added to 40 μL of dH_2_O in a 96-well plate, to which 50 μL of 5 mM p-NPP solution (Sigmafast p-NPP) was added. Well-plates were incubated at room temperature for 60 minutes, protected from light, and the absorbance was measured at 405 nm using an atomic absorbance spectrophotometer (Wallac Victor, Perkin Elmer Life Science). ALP activity was quantified against a standard curve of 0–80 nM p-NPP, expressed as U/mL and then normalised to pgDNA to estimate ALP concentration per cell. DNA content of ccMSCs was determined using a Hoechst DNA assay to normalise the ALP activity. Briefly, 140 μL of cell lysate (described above) was mixed with 60 μL Hoechst 33258 (Sigma) working dye solution in a black 96-well plate. Hoechst 33258 (Sigma) working dye solution was prepared by adding 20 mL of Hoechst buffer (100 mM Tris, 2 M NaCl and 10 mM EDTA) to 30 ml of ddH_2_0 and 5 μL of 1mg/ml Hoechst dye, protected from light. The fluorescence was measured at an emission of 460 nm and excitation of 365 nm using an atomic absorbance spectrophotometer (Wallac Victor, Perkin Elmer Life Science). DNA content was determined by using a known amount of purified calf thymus DNA as the standard.

Mineralisation of ccMSCs was also quantified after 21 days by alizarin red staining, whereby the stain was extracted using cethylpyridium chloride and the solution was measured at 550nm using a spectrophotometer. ccMSCs were washed gently with PBS and fixed with 4% paraformaldehyde for 15 minutes. The mineralised matrix was quantified by Alizarin Red S staining methods [43]. Alizarin red S (1 mole) binds to two moles of calcium forming an Alizarin Red S-calcium complex. Briefly, samples were stained with 40 mM alizarin red S (pH 4.2) for 15 min at room temperature. Samples were rinsed gently three times with distilled H_2_O to remove any unbound solution and then quantified by incubation with preheated 10% (wt/vol) cethylpyridium chloride for 20 minutes on an orbital rotator (Gyro-rocker SSL3, Stuart Scientific). Absorbance was measured at 562 nm using an atomic absorbance spectrophotometer (Wallac Victor). Using a standard curve, the absorption reading was converted to calcium ion concentration per mL.

### Statistical Analysis

Gene expression experiments were conducted in triplicate, giving an n = 3 per group. Data are expressed as a mean ± standard error. For gene expression analyses, significance of differences between the groups were examined using the general linear method for one-way analysis of variance (ANOVA) with the addition of Tukey's correction for multiple comparison testing. For flow cytometry and biochemical analyses, experiments were conducted in triplicate and repeated, giving an n = 6 per group. Data are expressed as a mean ± standard deviation. Statistical differences between groups were determined using a two-way analysis of variance (ANOVA), with treatment and time as the independent factors, followed by a pair-wise multiple comparison procedure (Tukey’s HSD test) was used to test for significance. For all comparisons, the level of significance was p ≤ 0.05. (Minitab V.16).

## Results

### Heat-induced MLO-Y4 cell responses

**Fig 1:**
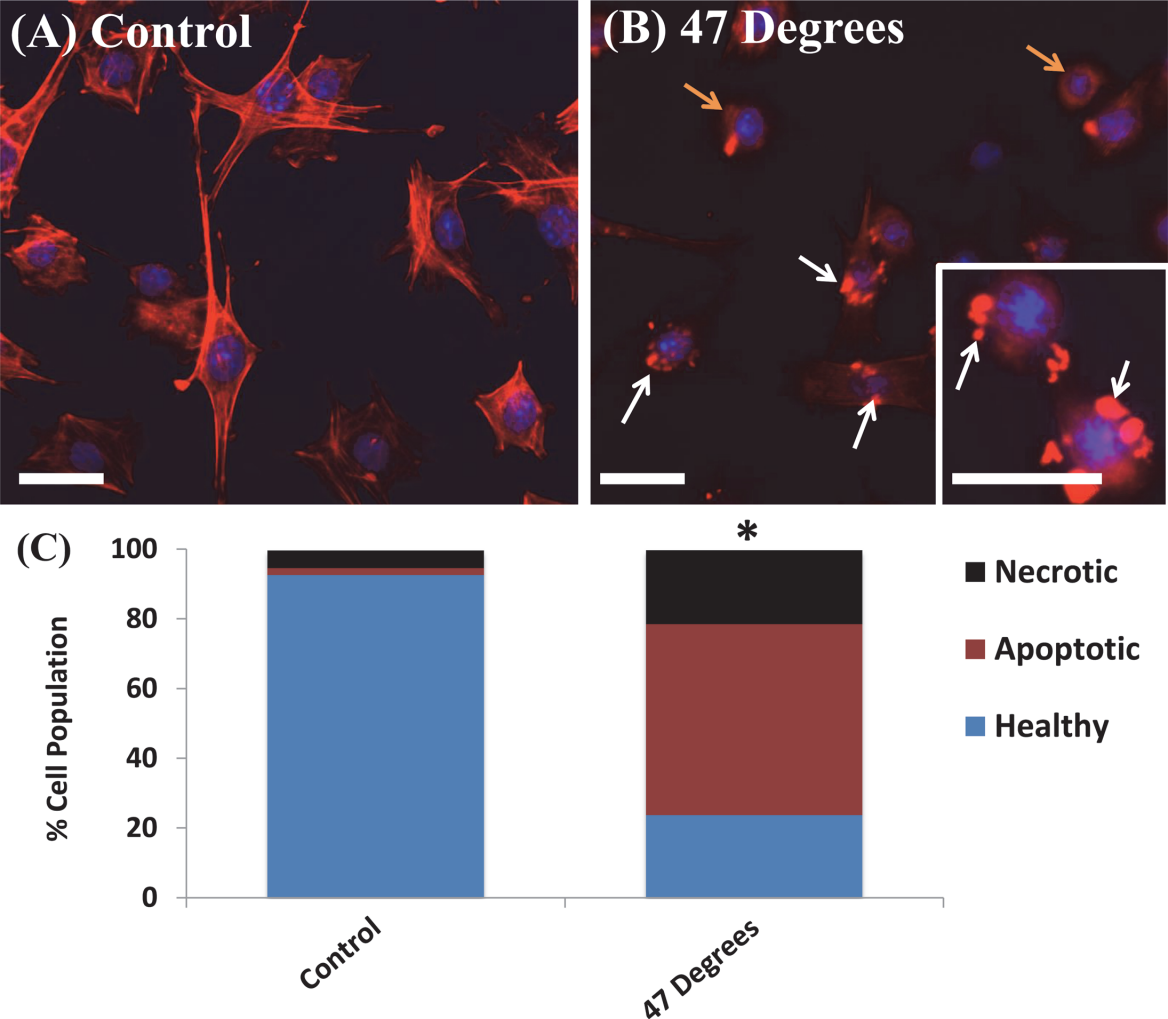
Heat-treatment induces damage responses in MLO-Y4 cells. Phalloidin stained actin filaments (red) and DAPI stained nucleus (blue) of MLO-Y4 cells heat-treated to (A) 37°C (control) and (B) 47°C for 1 minute demonstrating membrane condensation (white arrows) and rounded cell bodies detaching (orange arrows). Scale bar = 32μm. (C) Flow cytometry quantification of necrotic, apoptotic and healthy cell populations 24 hours after heat-treatment. * indicating statistical difference in the number of viable, apoptotic and necrotic cells compared to the 37°C (control) (p ≤ 0.05).

The effects of thermal elevation on cell morphology and structure, after 24 hours recovery, can be seen clearly in Fig. 1 (A). MLO-Y4s cultured at 37°C (control) show distinct actin filaments forming the cell cytoskeleton, characteristic of healthy cells. Cells exposed to 47°C for 1 minute show a less healthy cell population with areas of highly concentrated staining in the cytoskeleton (white arrows), which suggests condensation of the cytoskeleton and cell membrane, with some visible cells detaching and rounded cell bodies (orange arrows), characteristic of an unhealthy/dying cells.

Flow cytometry results reveal that, 24 hours after exposure to 47°C for 1 minute, the majority of the MLO-Y4 cell population (54.8%) is apoptotic, see Fig. 1 (B). A significant decrease in the number of viable cells (p = 0.0001), and a significant increase in the number of apoptotic (p = 0.0001) and necrotic (p = 0.0001) MLO-Y4s is seen 24 hours after exposure to 47°C for 1 minute, compared to the 37°C control.

### Gene Expression by Heat-Treated MLO-Y4 cells

**Fig 2:**
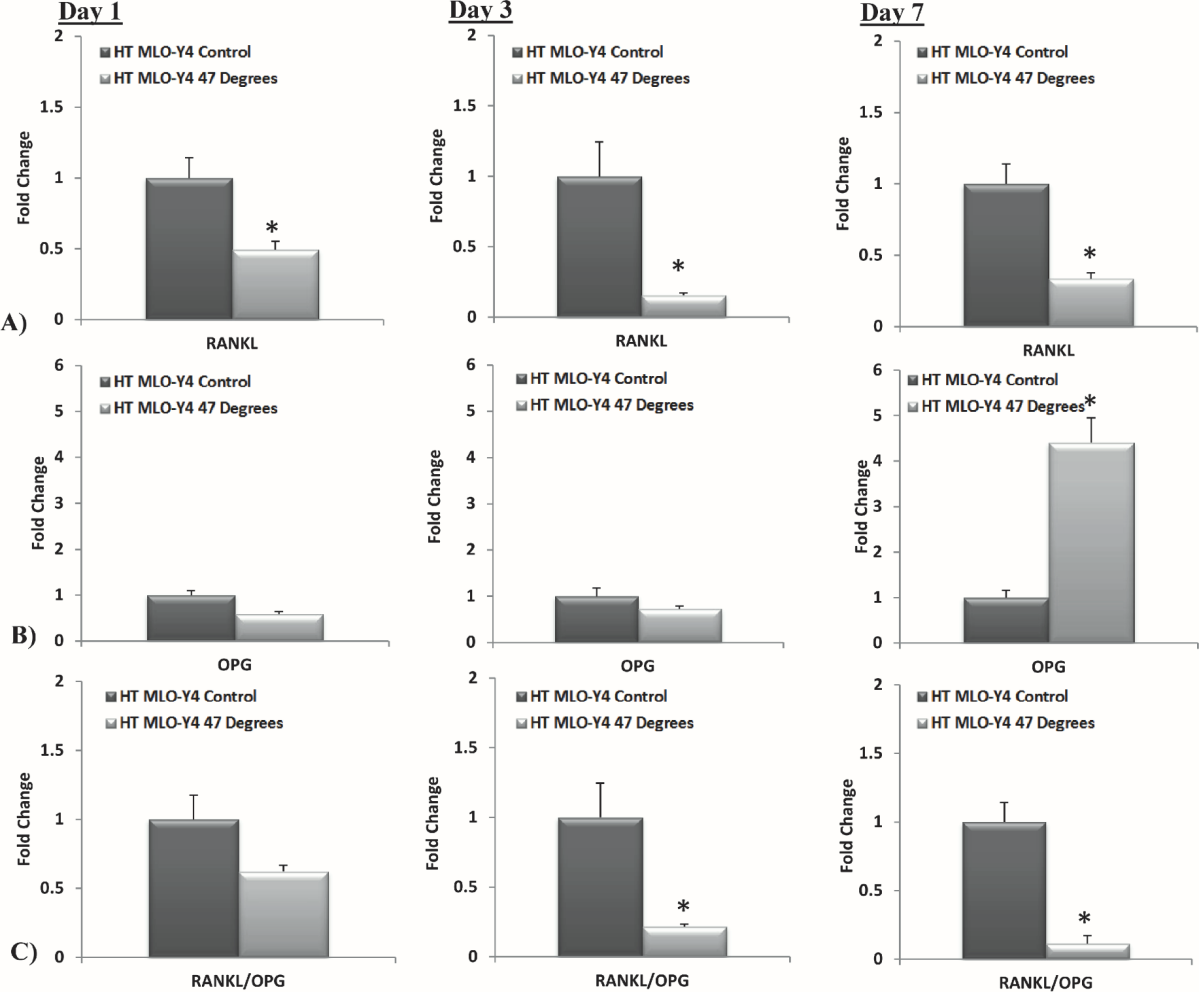
Heat-treatment of MLO-Y4 cells causes a down-regulation in the *Rankl/Opg* ratio. A) *Rankl*, B) *Opg* and C) *Rankl*/*Opg* gene expression by heat-treated (HT) MLO-Y4 cells to 47°C compared to the 37°C control group at 1, 3 and 7 days after heat-treatment (p≤0.05).

The results of the *Rankl* and *Opg* gene expression of heat-treated MLO-Y4s compared to the control are seen in Fig. 2. *Rankl* is a necessary factor for osteoclastogenesis [33,34], while the *Rankl* inhibitor *Opg* inhibits osteoclast differentiation [35,36]. An increase in the *Rankl*/*Opg* ratio promotes osteoclast differentiation while a decrease reduces mature osteoclast concentration [36,37,38,39]. A significant decrease in *Rankl* gene expression is seen in MLO-Y4 cells heat-treated to 47°C by day 1 (p = 0.0001), this decrease is maintained at days 3 (p = 0.0001) and 7 (p = 0.0011), see Fig. 2(A). No significant difference in *Opg* expression is seen in MLO-Y4 cells heat-treated to 47°C by day 1 and day 3, however by day 7 *Opg* expression is significantly increased compared to the control (p = 0.0006), see Fig. 2(B). The *Rankl*/*Opg* ratio is consistently down-regulated at days 1, 3 and 7 in MLO-Y4 cells heat-treated to 47°C compared to the control, significantly so at day 3 (p = 0.0150) and day 7 (p = 0.0073), see Fig. 2(C).

**Fig 3:**
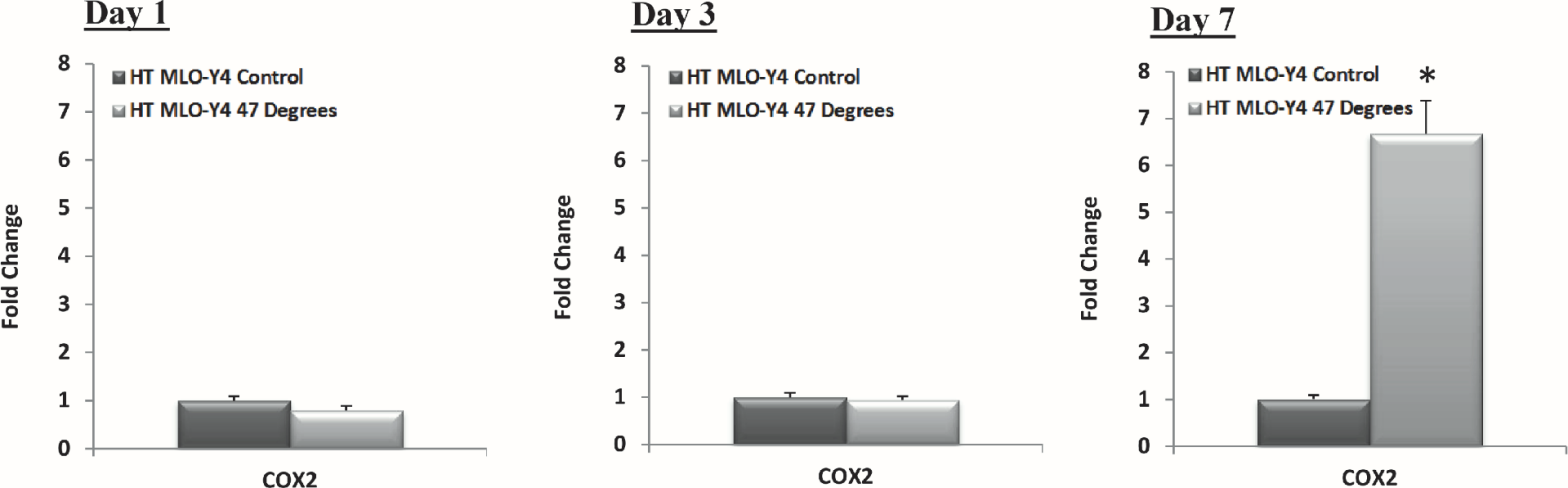
Heat-treatment of MLO-Y4 cells causes a late stage up-regulation in *Cox2* expression. *Cox2* gene expression of heat-treated (HT) MLO-Y4 cells (47°C) compared to the 37°C control group at, 1, 3 and 7 days after heat-treatment (p≤0.05).

The results of the *Cox2* gene expression, a vital signalling factor for osteogenic differentiation [40,41], of heat-treated MLO-Y4s compared to the control are seen in Fig. 3. No significant difference is seen in *Cox2* gene expression by day 1 and 3, however a significant increase is observed at day 7 in MLO-Y4 cells heat-treated to 47°C (p = 0.0009), compared to the control, see Fig. 3.

### Gene Expression by MLO-Y4s Co-Cultured with Heat-Treated MLO-Y4s

**Fig 4:**
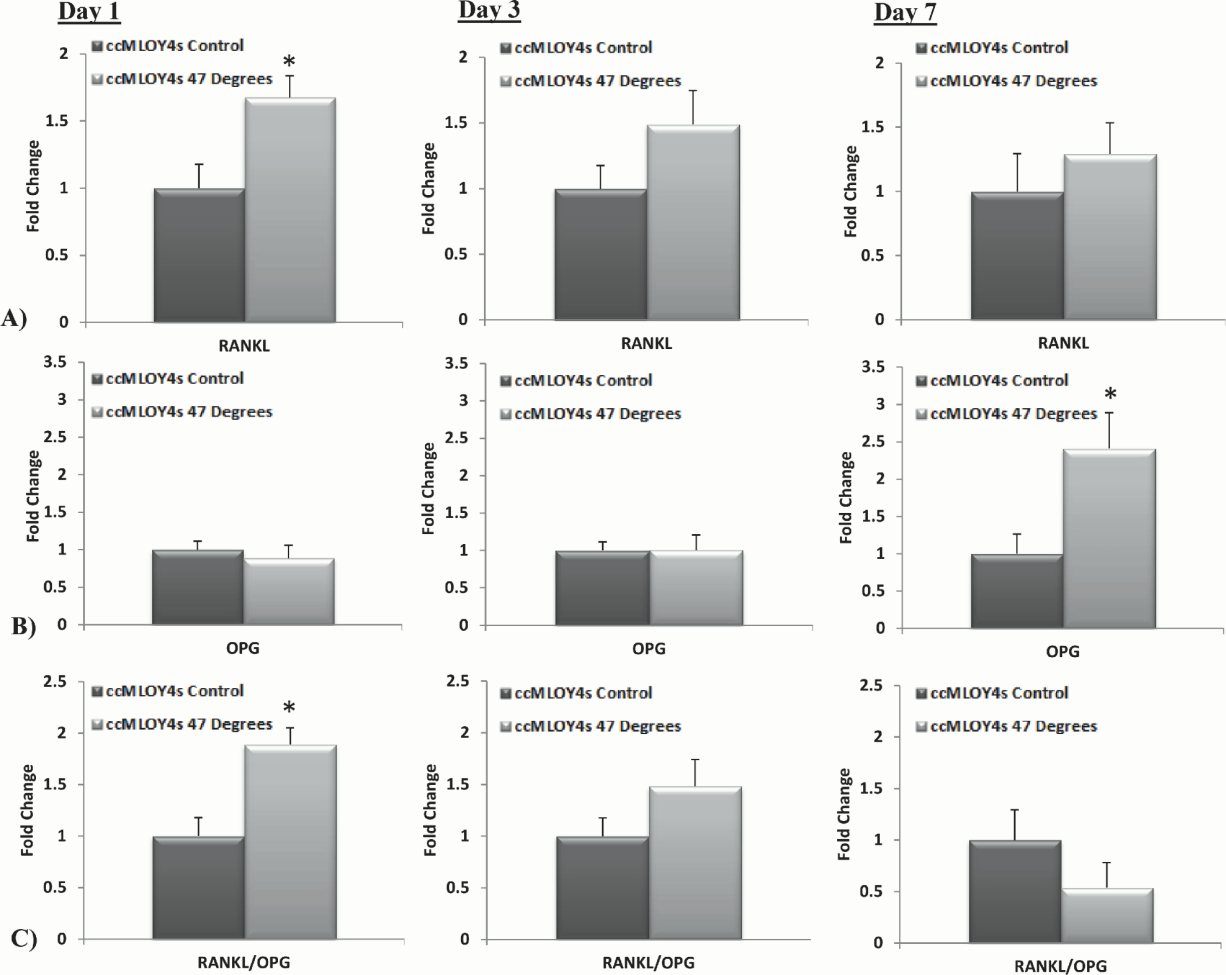
Co-culture of MLO-Y4 cells with heat-treated MLO-Y4s causes an initial up-regulation in the *Rankl/Opg* ratio. A) *Rankl*, B) *Opg* and C) *Rankl*/*Opg* gene expression by MLO-Y4 cells co-cultured (ccMLO-Y4s) with MLO-Y4 cells heat-treated to 47°C compared to the 37°C control groups at 1, 3 and 7 days after heat-treatment (p≤0.05).

The results of the *Rankl* (a vital signalling factor for osteoclastic differentiation [33,34]) and *Opg* (a soluble decoy receptor for *Rankl* [35,36]) gene expression of ccMLO-Y4s, co-cultured with the heat-treated MLO-Y4s, compared to the 37°C control are seen in Fig. 4. An initial (Day 1) significant increase in *Rankl* gene expression is seen in ccMLO-Y4 cells when cultured with MLO-Y4s that were heat-treated to 47°C (p = 0.00248). There was no significant difference in *Rankl* gene expression compared to control levels at day3 and day 7, see Fig. 4(A). No significant difference in *Opg* expression is seen in ccMLO-Y4 co-cultured with MLO-Y4s heat-treated to 47°C by day 1 and day 3, however by day 7 *Opg* expression is significantly increased compared to the control (p = 0.0066), see Fig. 4(B). The *Rankl*/*Opg* ratio is significantly up-regulated at day 1 (p = 0.0385) in ccMLO-Y4s cultured with MLO-Y4s that were heat-treated to 47°C compared to the control, see Fig. 4(C). The *Rankl*/*Opg* ratio was not significantly different control levels by day 3 and day 7.

**Fig 5:**
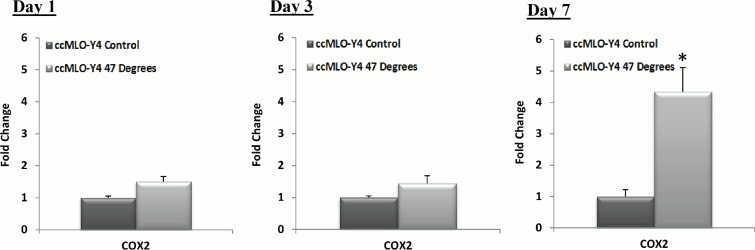
Co-culture of MLO-Y4 cells with heat-treated MLO-Y4s causes a late stage up-regulation in *Cox2* expression. *Cox2* gene expression by MLO-Y4 cells co-cultured (ccMLO-Y4s) with MLO-Y4 cells that were heat-treated to 47°C compared to the 37°C control group at 1, 3 and 7 days after heat-treatment (p≤0.05).

The results of the *Cox2* gene expression, a vital signalling factor for osteogenic differentiation [40,41], by ccMLO-Y4 cells, which were co-cultured with MLO-Y4 cells heat-treated to 47°C, compared to the 37°C control group are seen in Fig. 5. There is no significant difference in *Cox2* gene expression by day 1 and 3, however a significant increase is observed by day 7 (p = 0.0020), compared to the control, see Fig. 5.

### Osteoblastic Differentiation by Balb/c MSCs Co-Cultured with Heat-Treated MLO-Y4 Cells

**Fig 6:**
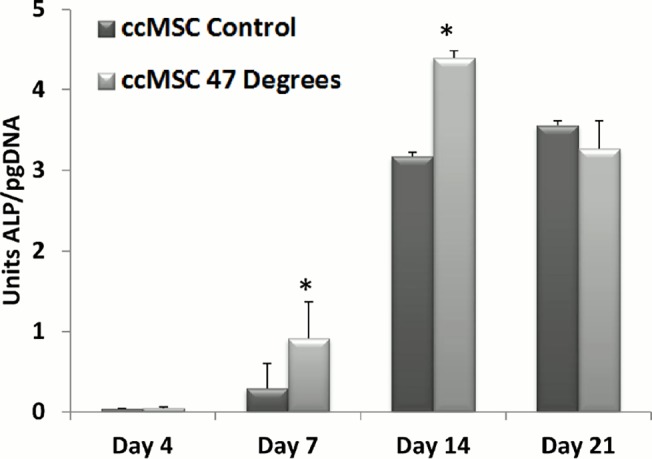
Co-culture of MLO-Y4 cells with heat-treated MLO-Y4s increases Alkaline Phosphatase Expression. Alkaline phosphatase activity of pre-osteoblastic MSCs 0, 4, 7, 14 and 21 days after their addition to MLO-Y4s heat-treated to 47°C, compared to the 37°C control (p≤0.05).

**Fig 7:**
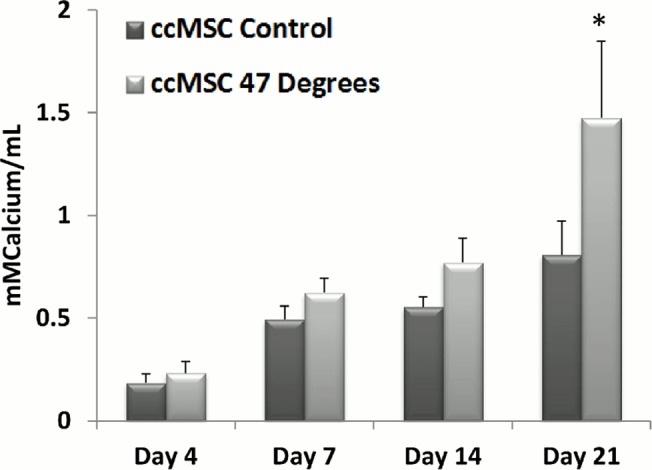
Co-culture of MLO-Y4 cells with heat-treated MLO-Y4s increases Calcium Deposition. Calcium deposition of pre-osteoblastic MSCs 0, 4, 7, 14 and 21 days after their addition to MLO-Y4s heat-treated to 47°C, compared to the control 37°C (p≤0.05).

The results of alkaline phosphatase activity and calcium deposition (markers of osteoblastic differentiation) of ccMSCs that were cultured with MLO-Y4s, which had been heat-treated to 47°C, compared to the 37°C control are seen in Figs. 6 and 7. A significant increase in ALP expression per picogram DNA is seen in ccMSCs, 7 days (p = 0.0032) and 14 days (p = 0.0001) after their addition to MLO-Y4s heat-treated to 47°C for 1 minute, compared to the 37°C control. There is no significant difference in ALP expression by day 21, see Fig. 6.

Calcium deposition is significantly higher in ccMSCs exposed to the biochemical factors produced by MLO-Y4s heat-treated to 47°C for 1 minute by day 21 (p = 0.0001), compared to the 37°C control, see Fig. 7.

## Discussion

This study identifies a link between osteocyte thermal damage and bone remodelling responses. Our results indicate that heat-treated osteocytes alter the expression of genes associated with both osteoclastogenesis and osteoblastogenesis in vitro. The pro-osteoclastogenic factor *Rankl* and the *Rankl*/*Opg* ratio is consistently down-regulated compared to the control at days 1, 3 and 7, while the *Rankl* competitor *Opg* is significantly up-regulated by day 7, in MLO-Y4s heat-treated to 47°C for 1 minute. Additionally, the pro-osteoblastogenic signaling factor *Cox2* is significantly up-regulated in heat-treated MLO-Y4 cells, 7 days after heat treatment. As such, the heat-treated MLO-Y4s are producing signals to initiate osteoblastic differentiation. Furthermore, we show that secreted factors from the heat-treated MLO-Y4 cells activate pre-osteoblastic Balb/c MSCs (ccMSCs) to increase production of the pro-osteoblastic differentiation marker, alkaline phosphatase after 7 and 14 days, as well as calcium deposition at day 21. Most interestingly, we also demonstrate a different response in nearby non-heat-treated MLO-Y4s (ccMLO-Y4s), whereby an initial (day 1) pro-osteoclastogenic signaling response (increased *Rankl* and the *Rankl*/*Opg* ratio). This is followed by later stage (day 7) pro-osteoblastogenic signaling, involving *Rankl* returning to control levels while *Opg* is significantly up-regulated, resulting in a down-regulation in the *Rankl*/*Opg* ratio. Additionally, by day 7 the pro-osteoblastogenic signaling marker *Cox2* is statistically up-regulated in ccMLO-Y4 that were cultured with MLO-Y4 cells heat-treated to 47°C. These results indicate that heat-treated osteocytes can elicit a remodelling response by signalling nearby MLO-Y4s, which in turn signal to activate pre-osteoclasts and pre-osteoblasts, as well as directly signaling to initiate nearby pre-osteoblastic MSCs to undergo osteogenic differentiation. Taken together, these results elucidate the effect of initial thermally induced osteocyte apoptosis to the remodelling responses in vitro, and as such identify for the first time the crucial role of the osteocytes in detecting and initiating the bone remodelling cascade in response to thermal damage.

The use of osteocyte-like MLO-Y4 cell-line is a possible limitation of this study with regard to interpreting the likely responses that would occur in human osteocytes in vivo. However, MLO-Y4s are widely used to study bone cell biology in vitro and have been shown to be suitable representatives of primary bone cells [11,25,26,27,30]. A second limitation of this study is that cells were cultured on tissue culture plastic, therefore organised two dimensionally, in monolayers, during the experimental studies. In vivo osteocytes are entombed in a three dimensional collagen and mineral matrix, and communicate with neighbouring osteocytes and osteoblasts via gap junctions at the cell processes [44,45,46,47]. However, this complex environment prevents direct study of the cell biology, and as such in vitro two dimensional cell culture permits simplification of this system to isolate and identify key signalling pathways. Moreover, our computational study confirmed that osteocytes experience the same temperatures as their surrounding matrix [8], and as such the temperature applied here is relevant for in vivo thermal elevations on osteocytes. A further limitation of this study is the use of a single temperature elevation criterion (47°C for 1 minute). Our previous study identifies the synergistic effect of the direct exposure of cells to this threshold temperature, eliciting osteocyte apoptosis, while also enhancing tissue regeneration by inducing differentiation and mineralised matrix production by osteoprogenitor cells [11]. Additionally, our computational study confirmed that osteocytes are likely to experience thermal elevations of 47°C for 1 minute during surgical orthopaedic cutting procedures [8], thereby validating the clinical relevance of this temperature elevation criterion for investigating thermally-induced responses of bone cells. A final limitation is that the in vitro co-culture technique utilised in this study is a simplified environment where the osteocyte-MSC relationship is investigated independently. The in vivo milieu is a complex signalling environment, which involves intercellular cross-talk between many cell types including osteoblast-lineage cells at all stages of differentiation, from pluripotent precursors to matrix-embedded osteocytes, and cells within the bone marrow, including adipocytes, T cells, and macrophages [44]. Co-culture [48,49] and conditioned media [14,48,50,51] approaches have been utilised to simplify this complex cellular environment. Most interestingly, Birmingham et al. reported higher mineralisation levels in osteocyte/MSC co-culture experiments, compared to the conditioned media approach. These results suggest that the physical presence of a directing cell type (osteocytes in this case), which are continually producing biochemicals, enhances the osteogenic response seen in MSCs, while the conditioned media approach will only provide snapshots of the biochemicals being produced and may omit vital signaling molecules/cytokines [48]. Using a co-culture approach, this study identifies the direct signalling contribution of thermally damaged osteocytes, and their resulting influence on nearby MSCs, in an effort to further the knowledge of this complex communication network.

Osteocytes are widely accepted as sensory cells that control and regulate bone remodelling in response to mechanical stimuli and micro-damage [52]. Previous studies have outlined that localised osteocyte apoptosis at damage sites plays a central role in ‘targeting’ remodelling [16,17,18,19,20,53]. Site-specific apoptotic death in response to micro-damage has been identified as the critical underlying mechanism by which micro-damage can be detected by neighbouring healthy osteocytes who have the capacity to produce essential cytokine signals (*Rankl*) to stimulate osteoclast differentiation [17,21,22,23]. In the current study we do not observe a pro-osteoclastogenic signalling response in MLO-Y4 cells directly exposed to 47°C for 1 minute at the time points analysed (1, 3 and 7 days). In fact, a down-regulation in *Rankl* and the *Rankl*/*Opg* ratio is observed at days 1, 3 and 7, together with an up-regulation in the *Rankl* competitor *Opg* and pro-osteoblastogenic gene *Cox2* at day 7, in the heat-treated group. However, pro-osteoclastogenic signalling (increased *Rankl* and the *Rankl*/*Opg* ratio) was in fact observed in MLO-Y4 cells that were co-cultured with heat-treated MLO-Y4s at day 1. Similar temporal changes in *Rankl* and *Opg* expression by osteocytes has been reported previously in response to micro-damage both in vitro (1–3 days) [21] and in vivo (3–7 days) [17,22], while temporal changes in *Cox2* expression have also been reported by MSCs and MC3T3s in response to mechanical stimulation (2–24 hours) in vitro [14]. In the current study the majority of the cell population directly exposed to thermal elevation (47°C for 1 minute) became apoptotic (54.8%), with a further 21.5% of the cell population necrotic, 24 hours after heat-treatment. Only 23.7% of the cell population remained healthy at this time-point. Recent studies have demonstrated that, in the case of micro-damage, osteocyte apoptosis is typically confined to discrete locations local to the micro-damage site and it is the nearby non-injured cells that are responsible for initiating pro-osteoclastogenic signalling [17,21], and the results of this study suggest that a similar response is initiated under thermally induced damage. Interestingly, we also observe pro-osteoblastic signalling from the MLO-Y4 cell population directly exposed to elevated temperatures (47°C for 1 minute) and resulting activation of nearby MSCs. Therefore, we propose that two discrete cell signalling populations may exist, whereby the apoptotic osteocytes are responsible for pro-osteoblastic signalling, whereas the nearby healthy osteocyte cells are responsible for both initial pro-osteoclastic and later stage pro-osteoblastic signalling. However, further in vivo studies are required to further understand the osteocyte signalling cascade in response to thermal damage and establish whether it is similar that mechanisms recently established for microdamage based remodelling.

A thorough biological understanding of cellular responses to thermal elevations is required to allow continual innovation of orthopaedic cutting techniques, as well as the use of thermally active materials and processes, and thermal therapies. In a previous study, using experimentally informed computational methods, we have outlined a range of temperatures likely to occur during orthopaedic cutting procedures [8]. Furthermore, we have predicted that embedded osteocytes experience thermal elevations of the surrounding matrix in which they are entombed, demonstrating that organ level thermal elevations are indeed felt by the embedded osteocytes [8], and as such, allow us to predict cellular level responses using the current in vitro approach. Due to their position embedded within the mineralised bone matrix, it is probable that osteocytes communicate with nearby MSCs exclusively through biochemical secreted factors such as *Rankl*/*Opg* and *Cox2*. The results of the current study suggest that the physical presence of osteocytes, which are continually producing biochemical signals in response to thermal damage, activate the neighbouring precursor cells. Therefore, we propose that heat exposure to osteocytes indeed triggers the bone remodelling process.

## Conclusion

In conclusion, the results of the current study provide a novel biological insight into the signalling response of bone cells from initial thermal damage, to the activation of the remodelling cascade in vitro. We identify a regenerative response by heat-treated MLO-Y4 cells, which is demonstrated by a consistent down-regulation in *Rankl* and the *Rankl*/*Opg* ratio at days 1, 3 and 7, together with an up-regulation in the *Rankl* competitor *Opg* and the pro-osteoblastogenic gene *Cox2* at day 7. We also observe an initial pro-osteoclastogenic signaling response, followed by later stage pro-osteoblastogenic signaling in non-heat-treated MLO-Y4s cells that are exposed to the biochemicals produced by the heat-treated MLO-Y4s. This is demonstrated by an initial increase in *Rankl*, and the *Rankl*/*Opg* ratio at day 1, followed by a down-regulation in *Rankl* and an up-regulation in *Opg* and *Cox2* by day 7. Furthermore, the pro-osteoblastic differentiation marker, alkaline phosphatase, and calcium deposition are increased in Balb/c MSCs by day 14 that were co-cultured with heat-treated MLO-Y4s. As such, this study identifies, for the first time, the critical role of the osteocytes in detecting and initiating the remodelling cascade in neighbouring cells in response to thermal damage. These results will inform the future development of orthopaedic cutting techniques that optimise thermal elevations in bone tissue, as well as the development of thermally active materials and thermal therapies for treatment of bone pathologies.

## Supporting Information

S1 Fig *Rankl* and *Opg* expression of heat-treated MLO-Y4 cells relative to day 1 control. A) *Rankl*, B) *Opg* and C) *Rankl/Opg* gene expression by heat-treated (HT) MLO-Y4 cells to 47°C compared to the 37°C control group at 1, 3 and 7 days after heat-treatment. * indicates statistical difference to all other groups, # indicates statistical difference to day 1 (p ≤ 0.05) and green broken line indicates day 1 control.
(TIF)

S2 Fig *Rankl* and *Opg* expression of co-cultured MLO-Y4 cells relative to day 1 control. A) *Rankl*, B) *Opg* and C) *Rankl/Opg* gene expression by MLO-Y4 cells co-cultured (CCMLO-Y4s) with MLO-Y4 cells heat-treated to 47°C compared to the 37°C control group at 1, 3 and 7 days after heat-treatment. * indicates statistical difference to all other groups (p ≤ 0.05) and green broken line indicates day 1 control.
(TIF)

S3 Fig *Cox2* expression of heat-treated and co-cultured MLO-Y4 cells relative to day 1 control. *Cox2* gene expression by (A) heat-treated (HT) MLO-Y4 cells (47°C) compared to the 37°C control, (B) MLO-Y4 cells co-cultured (ccMLO-Y4s) with MLO-Y4 cells that were heat-treated to 47°C compared to the 37°C control at 1, 3 and 7 days after heat-treatment. * indicates statistical difference to all other groups (p ≤ 0.05) and green broken line indicates day 1 control.
(TIF)
