# Supplementary figures and images for: Thermally Induced Osteocyte Damage Initiates a Remodelling Signaling Cascade
Source: PLoS One. 2015 Mar 18;10(3):e0119652. doi: 10.1371/journal.pone.0119652 (PMC4364670; doi:10.1371/journal.pone.0119652)

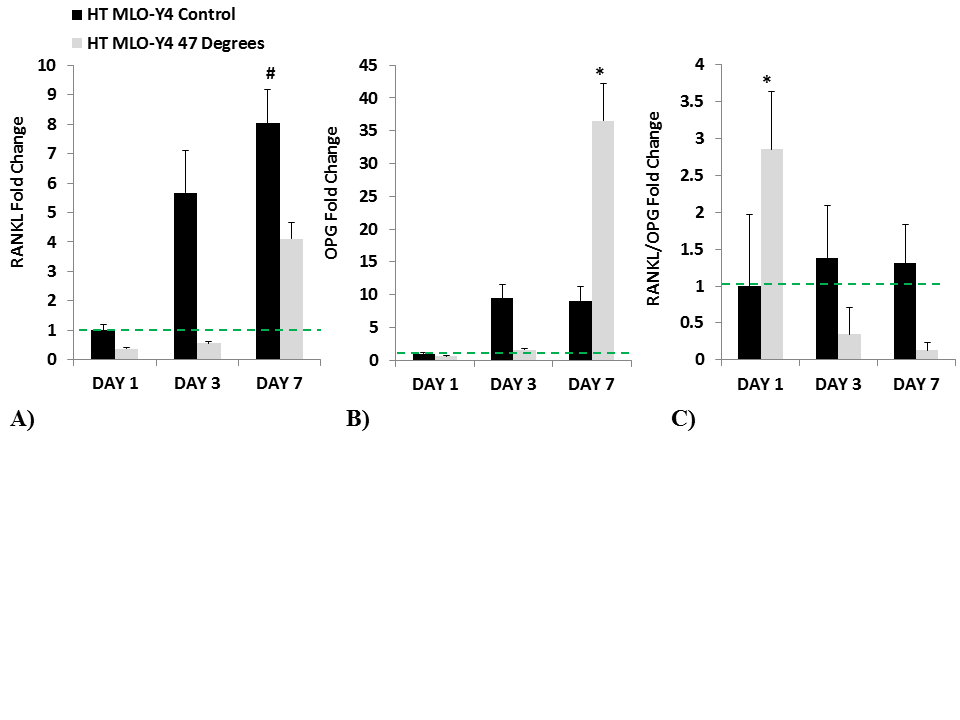

Supplement: S1 Fig — A) Rankl, B) Opg and C) Rankl/Opg gene expression by heat-treated (HT) MLO-Y4 cells to 47°C compared to the 37°C control group at 1, 3 and 7 days after heat-treatment. * indicates statistical difference to all other groups, # indicates statistical difference to day 1 (p ≤ 0.05) and green broken line indicates day 1 control. (TIF) [file pone.0119652.s001.tif]

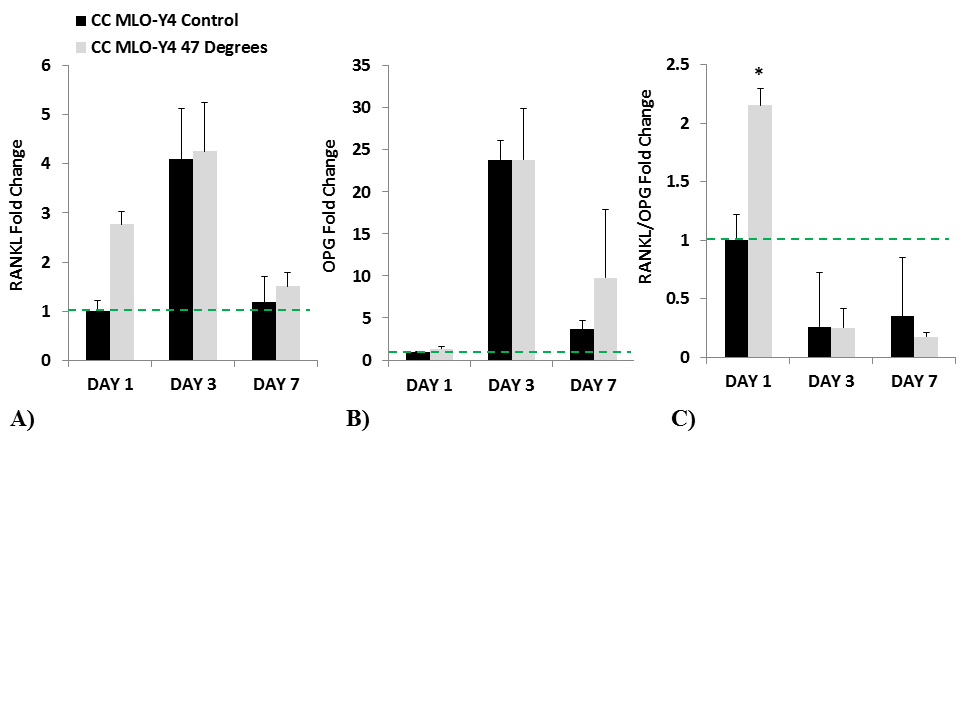

Supplement: S2 Fig — A) Rankl, B) Opg and C) Rankl/Opg gene expression by MLO-Y4 cells co-cultured (CCMLO-Y4s) with MLO-Y4 cells heat-treated to 47°C compared to the 37°C control group at 1, 3 and 7 days after heat-treatment. * indicates statistical difference to all other groups (p ≤ 0.05) and green broken line indicates day 1 control. (TIF) [file pone.0119652.s002.tif]

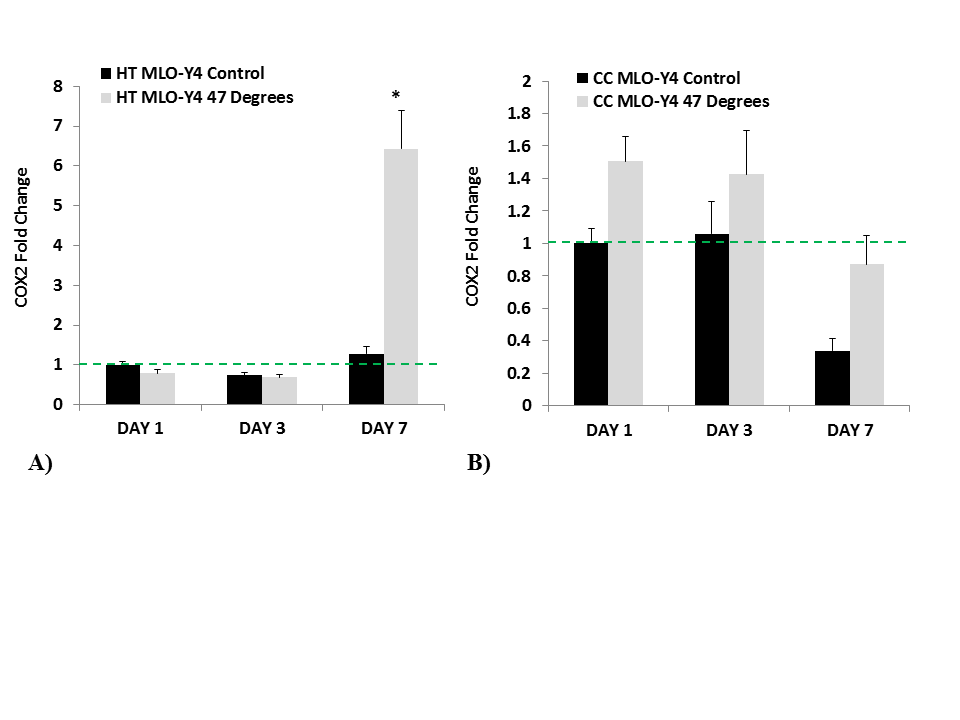

Supplement: S3 Fig — Cox2 gene expression by (A) heat-treated (HT) MLO-Y4 cells (47°C) compared to the 37°C control, (B) MLO-Y4 cells co-cultured (ccMLO-Y4s) with MLO-Y4 cells that were heat-treated to 47°C compared to the 37°C control at 1, 3 and 7 days after heat-treatment. * indicates statistical difference to all other groups (p ≤ 0.05) and green broken line indicates day 1 control. (TIF) [file pone.0119652.s003.tif]
